# Using a DNA mini-barcode within the ITS region to identify toxic* Amanita* in mushroom poisoning cases

**DOI:** 10.1007/s00253-024-13219-x

**Published:** 2024-06-17

**Authors:** Ran-Ran Xing, Wen-Ming Bai, Di Hu, Ting-Ting Deng, Jiu-Kai Zhang, Ying Chen

**Affiliations:** 1 National Key Laboratory for Market Supervision (Food Authentication), Chinese Academy of Inspection and Quarantine, No. 11, Ronghua South Street, Daxing District, Beijing, 100176 China; 2 College of Food Science, South China Agricultural University, Guangzhou, 510642 China; 3 Nanjing University of Finance and Economics, Nanjing, 210046 China

**Keywords:** *Amanita*, DNA mini-barcoding, Species identification, Internal Transcribed Spacer (ITS), Toxic mushroom

## Abstract

**Abstract:**

Mushroom poisoning contributes significantly to global foodborne diseases and related fatalities. *Amanita* mushrooms frequently cause such poisonings; however, identifying these toxic species is challenging due to the unavailability of fresh and intact samples. It is often necessary to analyze residues, vomitus, or stomach extracts to obtain DNA sequences for the identification of species responsible for causing food poisoning. This usually proves challenging to obtain usable DNA sequences that can be analyzed using conventional molecular biology techniques. Therefore, this study aimed to develop a DNA mini-barcoding method for the identification of *Amanita* species. Following the evaluation and optimization of universal primers for DNA mini-barcoding in *Amanita* mushrooms, we found that the internal transcribed spacer (ITS) gene sequence primer ITS-a was the most suitable DNA barcode primer for identifying *Amanita* species. Forty-three *Amanita* samples were subsequently amplified and sequenced. The sequences obtained were analyzed for intra- and inter-species genetic distances, and a phylogenetic tree was constructed. The findings indicated that the designed primers had strong universality among the *Amanita* samples and could accurately identify the target gene fragment with a length of 290 bp. Notably, the DNA mini-barcode accurately identified the 43 *Amanita* samples, demonstrating high consistency with the conventional DNA barcode. Furthermore, it effectively identified DNA from digested samples. In summary, this DNA mini-barcode is a promising tool for detecting accidental ingestion of toxic *Amanita* mushrooms. It may be used as an optimal barcode for species identification and traceability in events of *Amanita*-induced mushroom poisoning.

**Key points:**

• *Development of a DNA mini-barcoding method for Amanita species identification without fresh samples.*

• *The ITS-a primer set was optimized for robust universality in Amanita samples.*

• *The mini-barcode is suitable for screening toxic mushroom species in mushroom poisoning cases.*

**Graphical Abstract:**

**Figure:**
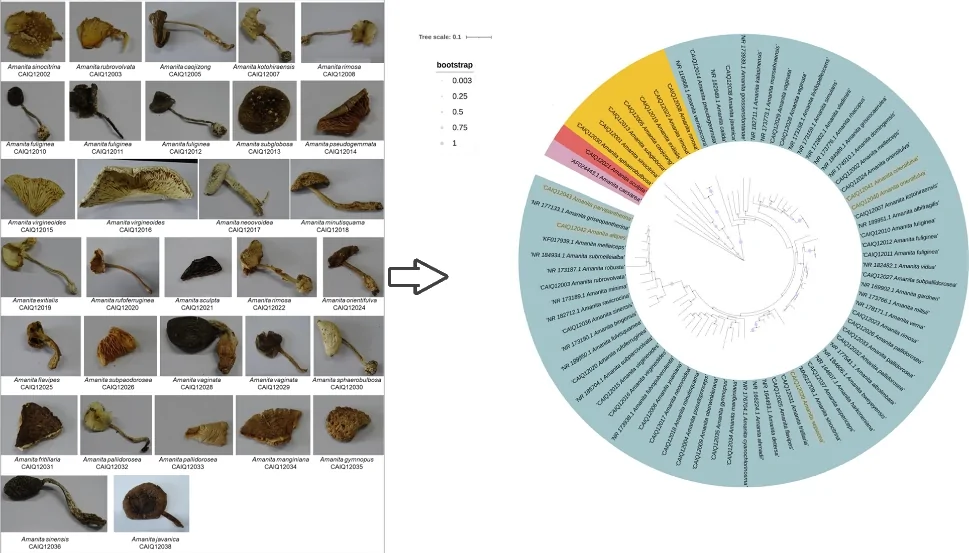

**Supplementary Information:**

The online version contains supplementary material available at 10.1007/s00253-024-13219-x.

## Introduction

Foraging and consuming wild mushrooms constitute a global tradition that is gaining momentum due to their harmonious alignment with prevalent dietary trends emphasizing natural, healthy, and sustainable choices. Nonetheless, it is imperative to note that mushroom poisoning stands as the foremost cause of foodborne disease outbreaks and associated fatalities on a global scale (White et al. 2019). China’s surveillance data from 2003 to 2017 indicate that mushroom intoxications constituted 31.8% of all recorded outbreaks and were responsible for 47.4% of the associated mortalities (Li et al. 2020). Moreover, from 2010 to 2020, the Chinese Center for Disease Control and Prevention (China CDC) reported 10,036 mushroom poisoning outbreaks, resulting in 38,676 illnesses and 788 deaths (Li, et al. 2021). In Germany, from 2000 to 2018, 4441 patients received hospital treatments due to the consumption of toxic mushrooms, resulting in 22 fatalities (Wennig et al. 2020). Additionally, Thailand reported 22,571 cases of mushroom poisoning and 106 associated deaths between 2003 and 2017 (Somrithipol et al. 2022).

Global inventory enumerates 140,000 distinct mushroom species. Among this diverse range, 5000 mushroom species are identified as toxic, with 100 species prominently implicated in most cases of mushroom poisoning (Dadpour et al. 2017). Most mushroom poisoning cases can be attributed to the ingestion of toxic mushrooms from the *Amanita* genus, which represents a significant part of the *Agaricales* order and *Amanitaceae* family (Diaz 2018). The *Amanita* genus comprises approximately 600 recognized agaric species worldwide. This includes both edible mushrooms such as *Amanita hemibapha*, *Amanita ovoidea*, and *Amanita caesarea* (Doğan 2013), as well as toxin-containing mushrooms including the notably highly toxic ones such as *Amanita subjunguilea*, *Amanita pallidorosea*, *Amanitin fuliginea*, *Amanita exitialis*, and *Amanitin rimosa* (Zhao et al. 2023). Within the *Amanita* genus, 37 cyclopeptide-containing *Amanita* species account for > 90% of all mushroom poisoning-related fatalities (Tang, et al. 2016). Inadvertent consumption of odorless and flavorless poisonous mushrooms is a concern, as many of these species contain potent toxins capable of causing severe illness or death if ingested (Vaagt et al. 2013).

Accurate identification of highly toxic mushroom species is important for effectively diagnosing and treating foodborne poisoning. Current methods for detecting toxic mushrooms primarily involve morphological identification, toxin analysis, and molecular detection. Traditional methods of species identification of mushrooms have relied on morphological characteristics. However, several species within *Amanita* genus exhibit significant morphological variation, for which there is no consensus on distinguishing characteristics. Furthermore, environmental conditions strongly influence morphological characteristics, making it difficult for untrained individuals to differentiate between toxic and edible mushrooms (Lima et al. 2012; Maeta et al. 2008). As a result, mushroom pickers might inadvertently ingest toxic wild mushrooms, leading to poisoning. Unfortunately, in most cases of mushroom poisoning, obtaining fresh and complete samples for identification is not feasible. Typically, samples are obtained from patient residues, vomit, or stomach contents, which complicates visual identification due to the compromised original morphological features. DNA barcoding, introduced in 2003, offers a precise and rapid alternative for species identification that minimizes human error (Hebert et al. 2003). Beyond its initial use, DNA barcoding has become a valuable tool for species identification and the monitoring of species diversity and abundance (Nehal et al. 2021). Notably, although DNA may undergo physical fragmentation during food processing and digestion, the genetic information carried by these sequences remains unchanged. This underscores the utility and robustness of DNA-based methods in identifying species from digested samples of toxic mushrooms (Garnica et al. 2016; Hebert et al. 2003).

The premise of DNA barcoding was that the sequence variation between species is greater than within species. Thus, this difference within a standardized region could be exploited for species-level identification. Researchers have successfully employed standard (full-length) DNA barcodes for identifying large fungal species and conduct molecular phylogenetic analyses (He et al. 2018; Zervakis et al. 2018). However, since the DNA is highly fragmented in cooked or digested samples, it is challenging to recover the complete DNA barcode information. To address this, a shorter gene region is required for species identification (Govender et al. 2019; Hajibabaei & McKenna 2012; Shokralla, et al. 2011). DNA mini-barcoding, a derivative method of DNA barcoding, can identify species in cooked or digested mushrooms, overcoming the limitations associated with full-length DNA barcoding (Xing et al. 2019). This approach addresses taxonomic and interspecific relationship issues using shorter target DNA sequences (100–300 bp) for species identification (Hajibabaei, et al. 2006; Hellberg and Morrissey 2011). The maturity of DNA mini-barcode technology has led to its wide use in identifying highly processed products from various plants, animals, and fungi (Govender, et al. 2019; Pan et al. 2020; Zhou, et al. 2023). Advances in molecular biology technologies and computer software facilitate the sequencing and comparison of DNA segments.

To diagnose and treat foodborne mushroom poisoning, it is crucial to accurately identify the highly deadly mushroom species (Zhou, et al. 2022). While some methods have contributed significantly to mushroom identification, there remains a need for more accessible techniques, particularly in the context of identifying toxic mushrooms. Our study addresses this need by proposing a DNA mini-barcoding approach that offers potential advantages over traditional methods. The present study aimed to evaluate and optimize the use of the DNA mini-barcoding method to identify the toxic *Amanita* species, which are frequently involved in such poisoning cases. The evaluated and examined DNA mini-barcode holds significant promise for analyzing long-term preserved *Amanita* specimens and samples retrieved after accidental ingestion of poisonous mushrooms. This method not only provides clinicians with a valuable tool for guiding medical treatment but also extends its utility to post-exposure forensic analysis. By aiding in the identification of the toxic mushroom involved, it becomes essential for the development of targeted treatments and informing public health interventions designed to prevent future poisonings. Moreover, the implications of this method reach beyond the clinical and forensic realms. It also serves as a foundation for creating educational initiatives that enhance public awareness about the hazards of toxic mushroom consumption. In conclusion, the evaluated DNA mini-barcoding method offers a multifaceted approach that addresses the needs of clinicians, forensic analysts, public health professionals, and the general public. Through its application, we aim to reduce the risks associated with mushroom poisoning and contribute to the overall safety of the food supply.

## Materials and methods

### Sample collection

**Fig. 1:**
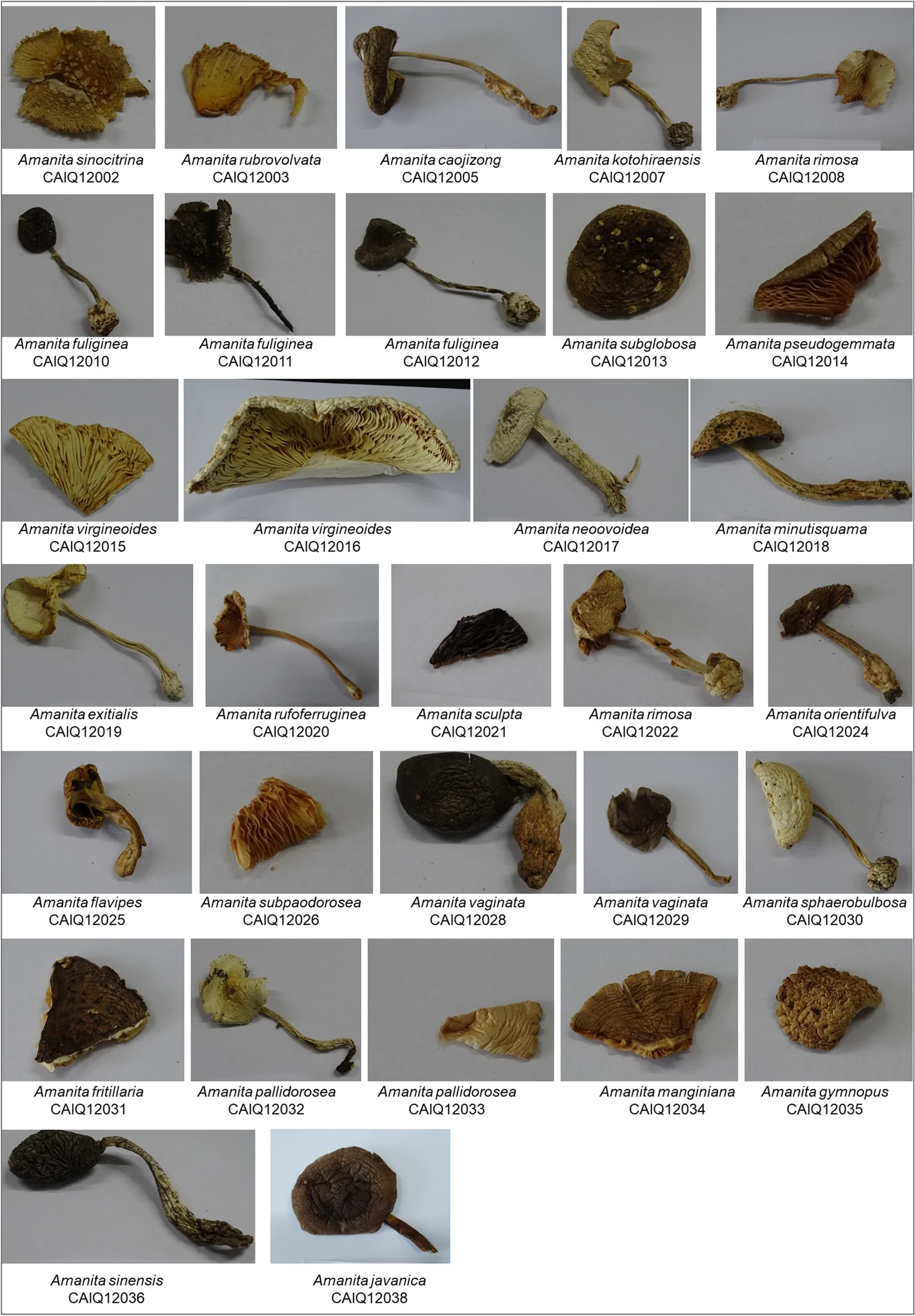
Morphological representation of the collected *Amanita* mushroom samples

A total of 43 wild mushroom samples, representing 33 species within the genus *Amanita,* were collected for this study (Table 1). All specimens of *Amanita* were identified using morphological characteristics, and the identification results were verified by full-length DNA barcoding with internal transcribed spacer (ITS), large subunit ribosomal ribonucleic acid (LSU rRNA), second-largest subunit RNA polymerase II (RPB2), and *β*-*tubulin* markers. In a previous study (Wenming, et al. 2021), we published the identification results based on full-length DNA barcoding (Table S1). Due to the scarcity of certain samples, it was unfeasible to provide a complete display of their morphological attributes. Consequently, Fig. 1 showcases a meticulously chosen set of 31 representative mushroom samples, characterized by relatively intact morphologies. The confirmed *Amanita* samples were utilized to optimize the designed mini-barcode primers.

**Table 1:** Mushroom species information and the identification results of 43 *Amanita* samples by DNA mini-barcoding

No	Sample ID	Sample species	Edibility	Mini-barcode results			
				GenBank Accession numbers			Length (bp)
				Top match	Similarity	Obtained in this study	
1	CAIQ12001	*Amanita sinocitrina*	Toxic	MN622709.1	100.00%	/	290
2	CAIQ12002	*A. melleiceps*	Toxic	KF017939.1	99.66%	PP256321	290
3	CAIQ12003	*A. rubrovolvata*	Toxic	JN943178.1	99.65%	PP256322	290
4	CAIQ12004	*A. pseudoprinceps*	Toxic	NR_159588.1	99.31%	PP256341	290
5	CAIQ12005	*A. caojizong*	Edible	NR_159571.1	99.62%	PP256387	290
6	CAIQ12006	*A. yuaniana*	Edible	MH508654.1	100.00%	PP262180	290
7	CAIQ12007	*A. kotohiraensis*	Toxic	MH508414.1	100.00%	PP262561	290
8	CAIQ12008	*A. rimosa*	Toxic	MN061275.1	99.31%	PP256489	290
9	CAIQ12009	*A. oberwinklerana*	Toxic	MH508446.1	100.00%	PP262572	290
10	CAIQ12010	*A. fuliginea*	Toxic	MN061271.1	100.00%	PP256335	290
11	CAIQ12011	*A. fuliginea*	Toxic	MW669576.1	100.00%	PP256336	290
12	CAIQ12012	*A. fuliginea*	Toxic	KU356799.1	100.00%	PP256337	290
13	CAIQ12013	*A. subglobosa*	Toxic	KF017947.1	100.00%	PP256494	290
14	CAIQ12014	*A. pseudogemmata*	Toxic	MK239258.1	100.00%	PP256502	290
15	CAIQ12015	*A. virgineoides*	Toxic	KP004958.1	98.97%	PP265498	290
16	CAIQ12016	*A. virgineoides*	Toxic	MG383668.1	100.00%	PP265111	290
17	CAIQ12017	*A. neoovoidea*	Toxic	MK279377.1	100.00%	PP265112	290
18	CAIQ12018	*A. minutisquama*	Toxic	NR_159582.1	100.00%	PP265113	290
19	CAIQ12019	*A. exitialis*	Toxic	OQ983896.1	99.66%	PP265114	290
20	CAIQ12020	*A. rufoferruginea*	Toxic	KU497532.2	100.00%	PP265115	290
21	CAIQ12021	*A. sculpta*	Toxic	N/A		PP265116	257
22	CAIQ12022	*A. rimosa*	Toxic	MN061275.1	99.31%	PP265117	290
23	CAIQ12023	*A. rimosa*	Toxic	N061275.1	100.00%	PP265118	290
24	CAIQ12024	*A. orientifulva*	Toxic	FJ441035.1	100.00%	PP265119	290
25	CAIQ12025	*A. flavipes*	Toxic	MH508346.1	100.00%	PP265120	290
26	CAIQ12026	*A. pallidorosea*	Toxic	MH508484.1	100.00%	PP256623	290
27	CAIQ12027	*A. subpallidorosea*	Toxic	KU601410.1	100.00%	PP256572	290
28	CAIQ12028	*A. vaginata*	Toxic	KF017948.1	100.00%	PP256586	290
29	CAIQ12029	*A. vaginata*	Toxic	AB458889.1	99.66%	PP256585	290
30	CAIQ12030	*A. sphaerobulbosa*	Toxic	N/A		/	289
31	CAIQ12031	*A. fritillaria*	Toxic	MK239240.1	100.00%	PP265121	290
32	CAIQ12032	*A. pallidorosea*	Toxic	KY626178.1	99.31%	PP265122	290
33	CAIQ12033	*A. pallidorosea*	Toxic	KY626178.1	99.31%	PP265122	290
34	CAIQ12034	*A. manginiana*	Edible	KT779083.1	100.00%	PP265123	290
35	CAIQ12035	*A. gymnopus*	Toxic	MH508393.1	100.00%	PP270376	290
36	CAIQ12036	*A. sinensis*	Edible	MK239263.1	100.00%	PP265124	290
37	CAIQ12037	*A. aspericeps*	Edible	MH508257.1	100.00%	PP265125	290
38	CAIQ12038	*A. javanica*	Toxic	MF421101.1	100.00%	PP265126	290
39	CAIQ12039	*A. sepiacea*	Toxic	KU139516.1	100.00%	/	290
40	CAIQ12040	*A. orientifulva*	Toxic	MH508467.1	100.00%	/	290
41	CAIQ12041	*A. orientifulva*	Toxic	MH508467.1	100.00%	/	290
42	CAIQ12042	*A. altipes*	Toxic	MH508254.1	100.00%	/	290
43	CAIQ12043	*A. parvipantherina*	Toxic	KF651008.1	100.00%	/	290

“N/A” indicates that sequences for the species are not available in GenBank databases. “/” indicates that the obtained sequences have not been submitted to GenBank databases

### In vitro digestion experiment to obtain degraded *Amanita* samples

To assess the capability of the DNA mini-barcode to identify degraded samples, a total of five samples representing *Amanita sepiacea*, *Amanita orientifulva* (2 samples), *Amanita altipes*, and *Amanita parvipantherina* were selected for a static in vitro digestion experiment. The in vitro digestion was performed following the procedure described by Minekus et al. (2014). To prepare the simulated oral fluid, a solution comprising 0.05 g KCl, 0.01 g CaCl_2_, 0.04 g KH_2_PO_4_, and 0.05 g NaHCO_3_ were dissolved in 30 mL distilled water. Subsequently, alpha-amylase (1.875 g) was added, and the pH was adjusted to 7.0 using 0.1 mol/L HCl. The simulated gastric fluid was prepared by dissolving 0.05 g KCl and 0.42 g NaCl in 70 mL distilled water. Pepsin (0.32 g) was added to the solution, and the pH was adjusted to 2.0 using 2 mol/L HCl. The volume was measured using a 100 mL volumetric flask and stored at 4 °C.

To simulate in vitro static digestion of the *Amanita* samples, 90 mg of each sample was weighed and placed in separate centrifuge tubes. The samples were then incubated in 5 mL of water at 37 °C for 2 h, in accordance with established protocols outlined in Minekus et al. (2014). The resulting solution was centrifuged, and the precipitate was collected in three aliquots (numbered 1–3). Aliquot 1 was mixed with 1 mL of distilled water, heated at 100 °C for 15 min, and then cooled to room temperature. Following this, the mixture was centrifuged at 4000 rpm for 5 min, and the resulting precipitate was collected. Aliquots 2 and 3 were each mixed with 1 mL of simulated oral fluid and incubated in a water bath at a constant temperature of 37 °C for 90 s with oscillation at a rate of 300 rpm, simulating the effect of oral liquid. Aliquot 2 was centrifuged at 4000 rpm for 5 min, and the resulting precipitate was collected. Aliquot 3 was rapidly mixed with 2 mL of 2 mol/L HCl solution to adjust the pH to approximately 2.0. The mixture was then combined with 2 mL of simulated gastric fluid and vigorously shaken for 5 s at room temperature. It was then incubated in a water bath at 37 °C for 1 h with oscillation at 300 rpm. After centrifugation, the resulting precipitate was collected.

### Use of a modified plant kit for DNA extraction and analysis

A modified method using the DNAsecure Plant Kit DNA New Plant Kit (Root) (Tiangen Biotech Co., Ltd., Beijing, China) was used to extract DNA from raw and digested samples. Specifically, three replicates of 30 mg of each sample were swiftly frozen in liquid nitrogen and homogenized. This was achieved using Aliquot 1 solution, 6 μL of RNase enzyme, and two grinding beads in a tissue grinder for 2 min, with a 1-min pause every minute. After allowing it to stand for 5 min, the remaining extraction steps were conducted following the instructions outlined in the kit. Subsequently, the DNA concentration and purity were assessed by measuring the absorbance at 230 nm, 260 nm, and 280 nm using an ultraviolet–visible (UV–vis) spectrophotometer.

### Design of mini-barcode primer sets for the identification of *Amanita*

**Fig. 2:**
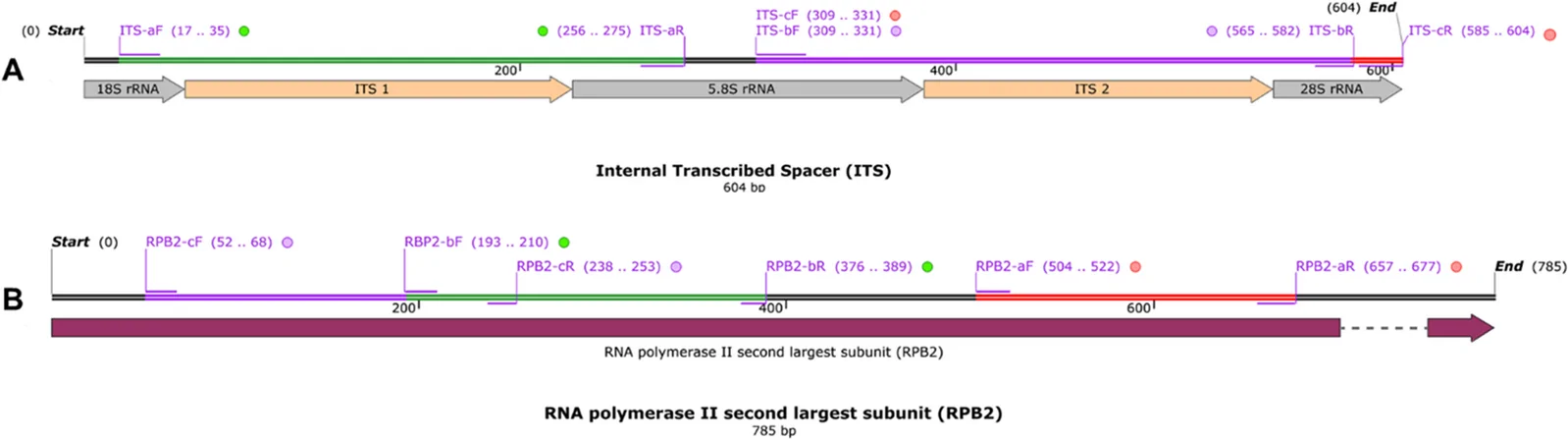
Schematic representation of amplified regions by the six designed mini-barcode primers, shown within the full-length RPB and ITS barcodes. Visualization of the RPB2 and ITS regions was performed using SnapGene Viewer v6.2.2 (http://www.snapgene.com/)

To facilitate the primer design for DNA mini-barcoding, we searched the NCBI GenBank database (https://www.ncbi.nlm.nih.gov/genbank/) using specific keywords “Amanita,” “RNA Polymerase II Second Largest Subunit,” and “Internal Transcribed Spacer” to retrieve the RPB2 and sequences for the *Amanita* genus. A total of 890 “RNA Polymerase II Second Largest Subunit” sequences and 6559 “Internal Transcribed Spacer” sequences were downloaded for analysis. The obtained sequences were aligned using the ClustalW tool implemented in the MEGA7 software (Kumar et al. 2016). We selected suitable conserved regions for primer design, ensuring that each had at least one conserved area upstream and downstream, with each conserved area comprising at least six amino acids. Ideally, the two conserved areas should be spaced 50 to 400 amino acid residues apart. We modified any misaligned bases using degenerate bases. When a conserved region of six amino acids was not present, we selected species with closer phylogenetic relationships for re-alignment. If conservation was still not met, a third round of alignment was performed, adjusting based on the previous results. Subsequently, mini-barcode primer sets were designed using Primer Premier 5.0 (Lalitha 2000). The designed primer sets were evaluated based on general principles of primer design, which include considerations for primer length, melting temperature (Tm), GC content, and avoidance of secondary structures such as hairpins, dimers, and repeats. These criteria ensure the specificity and efficiency of PCR amplifications (Dieffenbach et al. 1993). The characteristics of the primers were further assessed using Oligo 7.0. The specificity of the primers was verified through BLAST analysis (http://blast.ncbi.nlm.nih.gov/Blast.cgi). A total of six primer pairs were developed, consisting of three pairs for the RPB2 region (RPB2a, RPB2b, and RPB2c) and three pairs for the ITS region (ITS-a, ITS-b, and ITS-c). The primers positions of the RPB2 region and ITS region were visualized using SnapGene Viewer v6.2.2. (http://www.snapgene.com/), as shown in Fig. 2. The length and sequences of these primers are detailed in Table 2.

**Table 2:** Designed primers used for *Amanita* species identifications in this study

Gene fragments	Primer name		Primer sequence (5' → 3')	Primer length (bp)	Barcode length (bp)	References
RPB2	RPB2a	RPB2-aF	ATCYATGACGCCTGAAGAC	19	204	This study
		RPB2-aR	ATACTRGCACADATRCCRAGT	21		
	RPB2b	RBP2-bF	ATGGGYGTCCAYCGRGAT	18	200	This study
		RPB2-bR	CGCACRTGYTTCTTCTG	17		
	RPB2c	RPB2-cF	CTYGCRYTAATGGCTTGT	18	202	This study
		RPB2-cR	CCTTTCTCCGMAGTTT	16		
ITS	ITS-a	ITS-aF	TCCGTAGGTGAACCTGYGG	19	240	This study
		ITS-aR	GCTGYRTTCTTCATCGATGC	20		
	ITS-b	ITS-bF	AGTGAATCATCGARTBTTTGAAC	23	256	This study
		ITS-bR	AAGTTCAGCGGGTAGTCC	18		
	ITS-c	ITS-cF	AGTGAATCATCGARTBTTTGAAC	23	276	This study
		ITS-cR	TCCTCCGCYTWTTGATATGC	20		
	ITS4/5	ITS4	TCCTCCGCTTATTGATATGC	20	600 ~ 750	White et al. 1990
		ITS5	GGAAGTAAAAGTCGTAACAAGG	22		

### PCR amplification

PCR amplification was performed on the samples using the designed primers (Table 2). For the RPB2 sequences, a 25 μL reaction mixture comprising 2 × *Taq* PCR Master Mix (12.5 μL), 10 μM of each primer (0.3 μL), 10 ng/μL DNA template (2 μL), and sterile deionized water (9.9 μL). The PCR cycling conditions were as follows: 94 °C pre-denaturation for 5 min, denaturation at 94 °C for 30 s, annealing at 55 °C for 30 s, extension at 72 °C for 35 s, 35 cycles, and final extension at 72 °C for 10 min. For the ITS sequence, the 25 μL reaction mixture consisted of 2 × *Taq* PCR Master Mix (12.5 μL), 10 μM of each primer (0.3 μL), 10 ng/μL DNA template (2 μL), and sterile deionized water. The PCR cycling conditions were as follows: pre-denaturation at 95 °C for 5 min, denaturation at 95 °C for 45 s, annealing at 62 °C for 30 s, extension at 72 °C for 2 min, 30 cycles, and final extension at 72 °C for 10 min. The digested samples were also amplified using the universal primers ITS4/5 to amplify the full-length ITS barcode of 650 bp (White et al. 1990) (Table 2). The PCR products were visualized on a 2% agarose gel, and those with a single distinct band were purified and recovered.

### Sanger sequencing and data analysis

The PCR products were sequenced using BigDye® Terminator v3.1 and an ABI 3500 sequencer according to the manufacturer’s instructions. The nucleotide sequences were confirmed using SeqMan (DNASTAR Lasergene 7) and aligned using ClustalW in MEGA 7.0 (Kumar et al. 2016). The identity of amplified sequences was confirmed through BLAST analyses on GenBank. DNA sequences obtained using the ITS-a primer pair have been submitted to GenBank database, with the corresponding accession numbers detailed in Table 1. Neighbor-joining trees were constructed using MEGA 7.0 with Kimura 2-parameter distance. Bootstrap values above 95% were considered strongly supported.

## Results

### Optimizing universal primers for DNA mini-barcoding in *Amanita* mushrooms

This study designed six pairs of corresponding primers to amplify the short-length RPB2 and ITS mini-barcodes, which include three pairs each for the *RPB2* region (RPB2a, RPB2b, and RPB2c) and the ITS region (ITS-a, ITS-b, and ITS-c), as detailed in Fig. 2 and Table 2. The design of these primers took into consideration several factors including primer specificity, genetic diversity of the amplification region, and technical feasibility. For the ITS region, which is part of the ribosomal RNA comprising ITS1, 5.8S rRNA gene, and ITS2 (Schoch et al. 2012). ITS1 is located between the 18S and 5.8S rRNA genes, while ITS2 is situated between the 5.8S and 28S rRNA genes. The ITS-a primers were specifically engineered to amplify the ITS1 region, whereas the ITS-b and ITS-c primers target the ITS2 region. The selection and design of these primers were based on their specificity and amplification efficiency within these sub-regions, aimed at enhancing the accuracy and efficiency of species identification. For the *RPB2* region, the primers RPB2a, RPB2b, and RPB2c were designed to target coding regions devoid of introns. This strategic selection was informed by the gene’s characteristic of having a limited number of introns that are highly conserved across the genome, thereby enabling the generation of clean amplicons suitable for phylogenetic analysis (Matheny et al. 2007).

The amplification of the designed primers was performed using DNA samples from *Amanita caojizong* and *Amanita rubrovolvata* (Fig. S1). Among the three RPB2 primer sets, RPB2-b exhibited the ability to amplify two target bands within the range of 100–200 bp. Conversely, RPB2-a generated one distinct band, while RPB2-c did not yield corresponding bands. Nineteen *Amanita* samples (Table 1, Nos. 1–19) were amplified using the RPB2-b primer; all samples could produce a single band with a length of 100–200 bp, as shown in Fig. S2. PCR products were sequenced using Sanger sequencing. The findings showed that, except for *Amanita sinocitrina*, *Amanita fuliginea, Amanita rimosa*, and *Amanita flavipes*, which showed a sequence similarity of 99–100% on NCBI, the remaining *Amanita* species showed alignment results similar to *Amanita fuliginea*. Despite the technical success in amplification, the RPB2-b primer set exhibited limited discriminatory power, as the majority of the species displayed sequence alignment similar to *Amanita fuliginea*. This outcome suggests that the targeted region of the RPB2 gene may not possess sufficient variability to distinguish between closely related species within the genus *Amanita*.

The ITS primers ITS-a, ITS-b, and ITS-c successfully amplified the target bands ranging from 250 to 500 bp; however, they also exhibited nonspecific amplification. The ITS-a primer, which is relatively short in length, was chosen to optimize the amplification system and program resulting in an optimal system and program. Nineteen *Amanita* samples (Table 1, Nos. 1–19) were amplified, and all samples produced bands ranging from 250 to 500 bp. The PCR products were sequenced using Sanger sequencing, and 19 gene sequences of 230–290 bp were obtained. These sequences were compared against reference sequences in the NCBI database. The sequences from 19 *Amanita* species, including *Amanita sinocitrina*, *Amanita fuliginea*, *Amanita rimosa*, and *Amanita flavipes*, showed high similarity (99–100%) with the reference sequences in the NCBI database.

Thus, based on comprehensive considerations encompassing PCR amplification efficiency, sequencing success rate, and species identification accuracy, our findings indicate that the ITS gene sequence primer ITS-a emerges as the optimal candidate for DNA barcoding in subsequent *Amanita* species identification experiments. Consequently, we propose the adoption of ITS-a as the mini-barcode standard for *Amanita* genus.

### Assessment of the universality of DNA mini-barcode primers for *Amanita* species identification

**Fig. 3:**
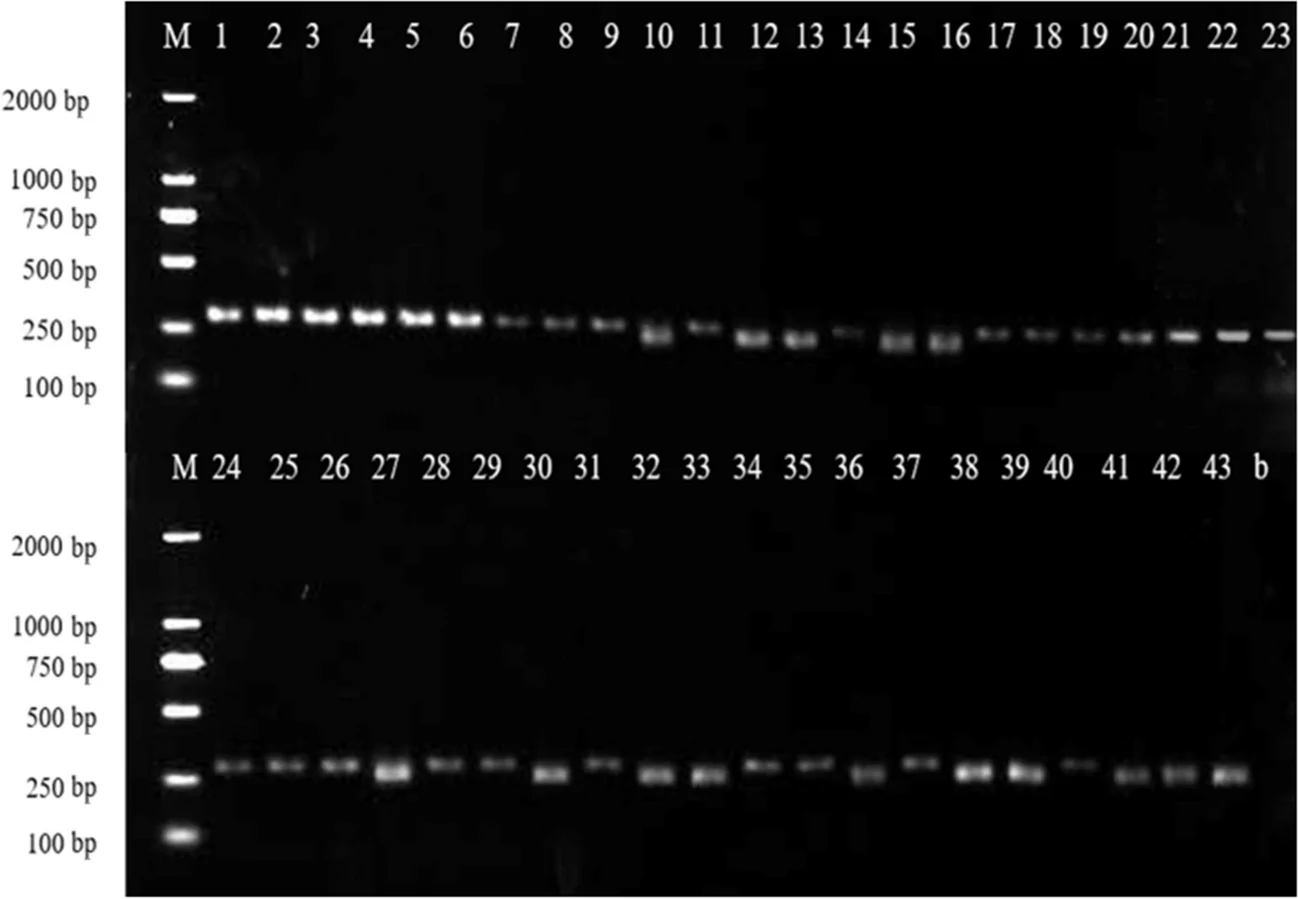
PCR electrophoresis analysis of ITS in *Amanita* samples using mini-barcode primers. M, marker; 1–43: *Amanita* samples corresponding to Nos. 1–43 in Table 1; b, blank control

Consuming toxic mushrooms leading to food poisoning incidents is a common occurrence, and among the primary causative agents is *Amanita*. Identifying poisonous mushroom species consistently revolves around this genus. To evaluate the universality of the ITS-a universal primer for PCR amplification across *Amanita* species, we used this designed primer to amplify ITS fragments from the genomic DNA extracted from 43 *Amanita* samples (Table 1). The DNA concentration and purity from each sample were determined using a Nanodrop ultra-micro spectrophotometer. All samples had DNA concentrations ranging from 40 to 584.8 ng/L and *A*_260_/*A*_280_ values ranging from 1.60 to 2.06, meeting the requirements for subsequent experiments. Electrophoresis results revealed that we achieved a 100% success rate (43/43) in amplifying the target gene fragment for all 43 *Amanita* samples using the designed universal primer. Notably, we observed single and bright bands, as depicted in Fig. 3, indicating that the designed universal primer had excellent universality in these experiments.

### Evaluation of the accuracy of DNA mini-barcoding for identifying *Amanita* species

We conducted a comprehensive assessment to determine the efficacy of using ITS mini-barcodes in identifying *Amanita* species. Through bidirectional sequencing with an ABI 3500 gene analyzer, followed by subsequent sequence splicing and proofreading, we acquired 43 DNA mini-barcode sequences. The sequencing success rate was 100%, with an average sequence length of 290 bp (except for CAIQ12021 and CAIQ12030) and a G + C content spanning from 33.33 to 45.52%. On average, the G + C content was calculated at 40.62%. Comparative analysis of the aligned DNA mini-barcodes against reference sequences from the GenBank database demonstrated a species-level identification accuracy exceeding 99%, except for *Amanita sculpta* (CAIQ12021) and *Amanita sphaerobulbosa* (CAIQ12030), exhibiting similarity levels lower than 91%. Interestingly, no sequences corresponding to these two *Amanita* species were found in the GenBank database. The comparison results of the 43 *Amanita* samples were consistent with our full-length DNA barcode identification methods (Wenming, et al. 2021), validating the discriminative capacity of the designed ITS mini-barcode. All nucleotide sequences were submitted to NCBI GenBank, and their accession numbers are listed in Table 1.

### Genetic variation and phylogenetic tree analysis of ITS mini-barcoding

As per the Consortium for the Barcode of Life (CBOL) recommendations, the K-2-P parameter model was applied in this study to calculate both the intra- and inter-specific genetic distances for the *Amanita* samples (Shen et al. 2016). The DNA mini-barcode ITS sequence analysis exposed a minimum inter-specific genetic distance of 0.036, whereas the maximum intra-specific genetic distance was observed to be 0.008. The average inter-specific genetic distance was 285 times higher than the average intra-specific distance, underscoring a marked divergence. The substantial difference between the minimum inter-specific distance and maximum intra-specific distance indicates the presence of a clear “Barcoding gap” with no overlap between intra- and inter-specific distances. This demonstrates the suitability of the ITS fragment for use as a DNA mini-barcode.

**Fig. 4:**
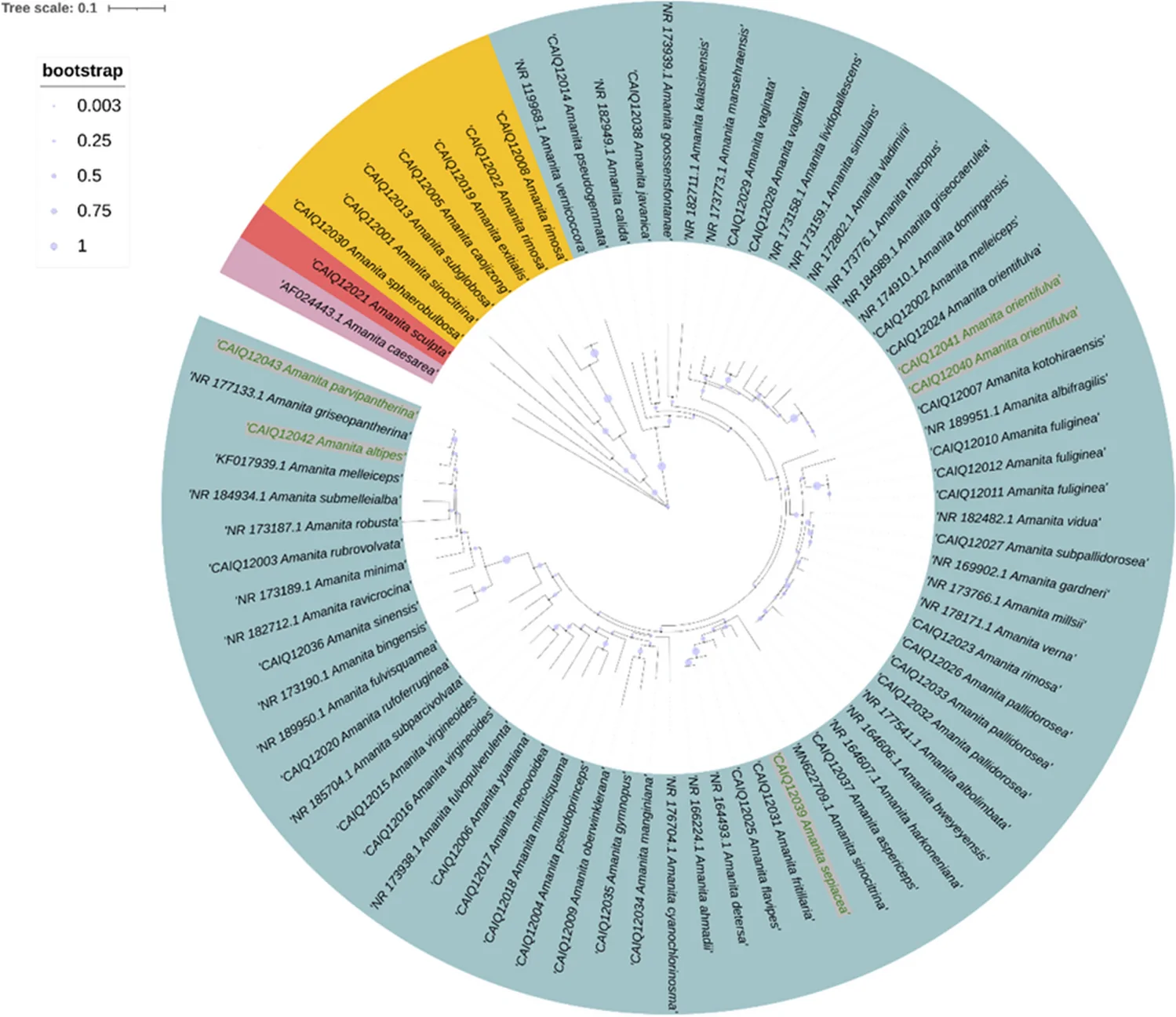
Neighbor-joining phylogenetic trees constructed based on DNA mini-barcodes for *Amanita* species. Different branches are annotated with distinct colors. Digested samples (CAIQ12039–CAIQ12043) are highlighted with green font on gray background

In this study, phylogenetic analysis was conducted utilizing the ITS mini-barcode derived from ITS gene sequences obtained from 43 samples of *Amanita*, along with 33 sequences sourced from the Fungal Internal Transcribed Spacer RNA (ITS) RefSeq Targeted Loci Project in the NCBI database (Accession: PRJNA177353 ID: 177353), with *Amanita caesareae* designated as the outgroup. The resulting NJ tree (Fig. 4) revealed a distinct clustering pattern, wherein *Amanita sculpta* appeared on a solitary branch, while *Amanita sphaerobulbos*a, *Amanita sinocitrina*, *Amanita subglobosa*, *Amanita caojizong*, *Amanita exitialis*, and *Amanita rimosa* formed a cohesive cluster on another branch. In contrast, the remaining *Amanita* species grouped into individual branches, each species forming its distinct family. This indicates that the ITS mini-barcode has high applicability and feasibility for species identification (Bagley et al. 2019). Additionally, sequences derived from digested samples were also aligned in the phylogenetic tree (highlighted with green font on gray background), demonstrating concordance with sequences from the same species in the phylogeny (such as *A. orientifulva*), thereby further validating their utility in resolving species relationships.

### Assessment of DNA mini-barcode primers for amplifying degraded *Amanita* DNA samples

**Fig. 5:**
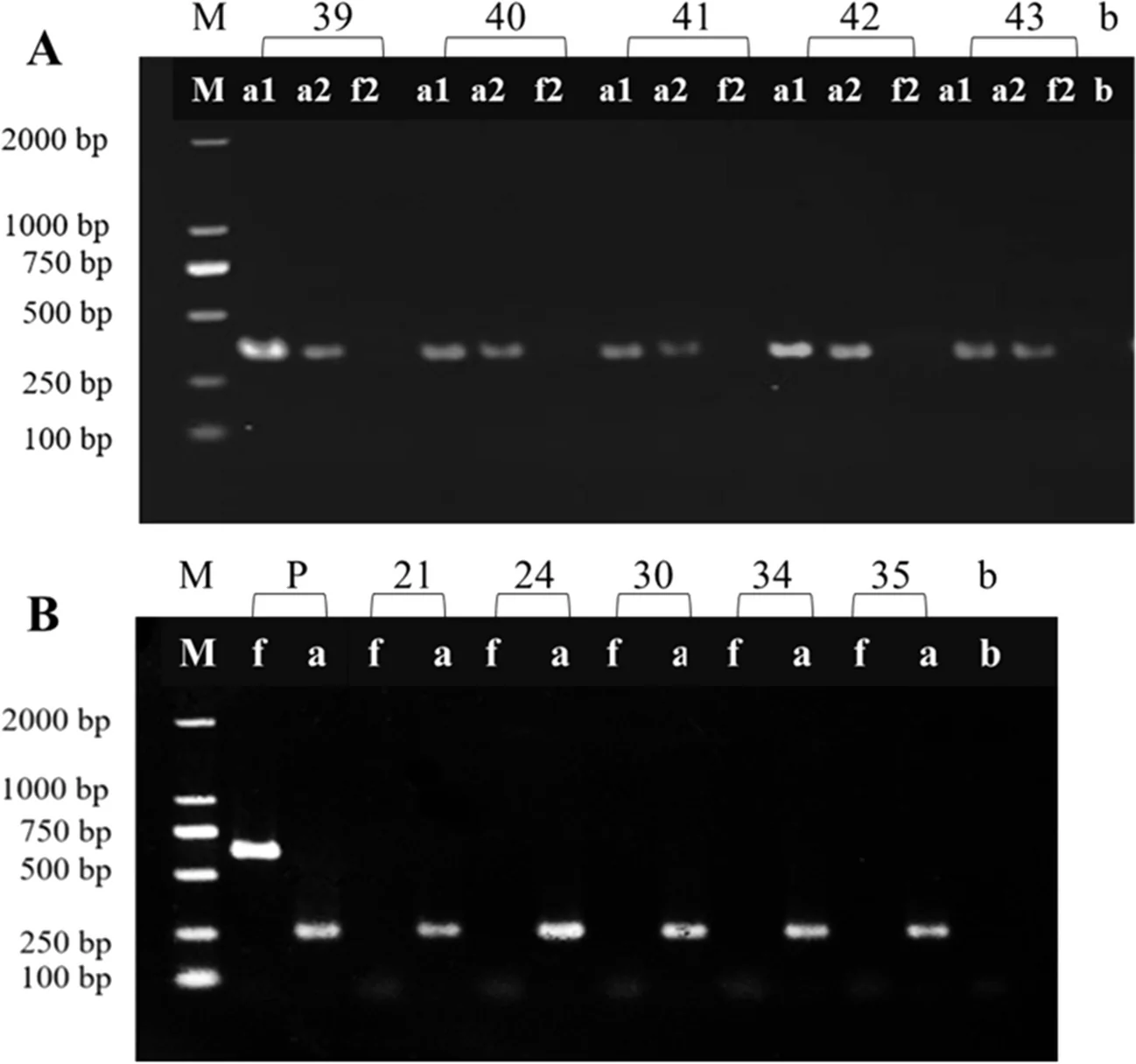
Electrophoresis visualization of PCR amplification using ITS mini-barcode and full-length ITS barcode for digested (**A**) and prolonged stored (**B**) *Amanita* samples. M, marker; a1, primer ITS-a for the pre-digestion sample; a2, primer ITS-a for the post-digestion sample; f2, primers ITS4/5 for the post-digestion sample; b, blank; P, positive control (*Amanita caojizong*); a, ITS-a primer; f, ITS4/5 primer; numbers correspond to sample no. Listed in Table 1

This study aimed to evaluate the efficacy of ITS mini-barcode primers in amplifying degraded DNA from mushroom samples subjected to simulated oral and gastric conditions, as well as from long-term storage. We selected five *Amanita* mushroom samples (Table 1, Nos. 39–43) for static simulated digestion experiments. DNA was extracted from samples subjected to three stages of complete digestion: boiling at 100 °C, simulated oral digestion, and simulated gastric digestion. The ITS-a primer was used to amplify the DNA mini-barcode shorter than 300 bp, while the ITS4/5 primers were used to amplify the full-length ITS barcode of 650 bp. Results revealed that the five *Amanita* mushroom samples subjected to static simulated digestion experiments failed to amplify the 650 bp full-length ITS barcode but successfully amplified DNA mini-barcodes of approximately 290 bp (Fig. 5A). This indicates the suitability of the ITS-a primer for PCR amplification of degraded DNA resulting from processing and digestion. These findings hold potential implications for using DNA mini-barcoding in the identification and analysis of *Amanita* species.

Additionally, due to prolonged storage, certain samples of *Amanita* experienced a degree of DNA degradation, such as *Amanita sculpta* (Table 1, CAIQ12021) *Amanita orientifulva* (Table 1, CAIQ12024), *Amanita sphaerobulbosa* (Table 1, CAIQ12030), *Amanita sphaerobulbosa* (Table 1, CAIQ12034), and *Amanita gymnopus* (Table 1, CAIQ12035) experienced significant DNA degradation due to extended storage periods. These five samples, previously shown in our previous study to be unable to amplify the 650 bp full-length ITS DNA barcode fragment using the ITS4/5 primers, were found in this study to successfully amplify a 290 bp ITS gene fragment (Fig. 5B). This indicates that the designed primers were effective in identifying samples with degraded DNA that had been stored for a prolonged period.

The study demonstrates the efficacy of the ITS-a primers in amplifying degraded DNA. It emphasizes the likelihood of DNA degradation in mushroom samples exposed to simulated oral and gastric conditions or extended storage. These findings highlight the potential of DNA mini-barcoding to identify and study mushroom species, with DNA integrity critically influencing result accuracy.

## Discussion

In this study, the challenges of identifying mushroom species in poisoning cases are compounded by the frequent unavailability of fresh, intact specimens. Data from the China CDC indicates that in 10,036 mushroom poisoning outbreaks, only 3872 (38.6%) specified the mushroom names, while 6164 (65.1%) involved fatalities linked to unidentified mushrooms. The absence of relevant samples and the ingestion of unknown mushrooms have considerably complicated the identification of causative species. Due to the unavailability of fresh and intact specimens in mushroom poisoning incidents, identification must rely on remnants, vomitus, or stomach aspirates from affected individuals. The severe degradation of samples results in highly fragmented and diluted DNA, presenting challenges in obtaining full-length DNA barcodes (Velasco-Cuervo et al. 2019). Therefore, under such circumstances, using shorter DNA mini-barcodes proves advantageous for species identification in the sample material (Maeta, et al. 2008).

In the realm of DNA barcoding, the challenges posed by DNA degradation and poor DNA quality are well-recognized during PCR amplification of full-length DNA barcodes. This hurdle is particularly pronounced in processed products and long-term preserved specimens. However, DNA barcoding remains an accurate and straightforward method for species identification. Both full-length DNA barcode and mini-barcode have been employed to identify species in processed products that have undergone DNA degradation or fragmentation. The past two decades have witnessed significant advancements in molecular techniques for fungal identification, leading to the widespread adoption of various DNA barcodes. Notably, the ITS (Justo et al. 2010; Tang et al. 2015), the LSU (Qing et al. 2012; Parnmen, et al. 2016), the RPB2 (Matheny et al. 2007; Li et al. 2017), and the translation elongation factor 1a (EF-1a) (Zhao et al. 2021) have become the most widely used barcodes for fungi identification.

In this study, we employed two widely recognized molecular markers in mycology: the protein-coding RPB2 gene and the ITS region of the ribosomal RNA gene locus, as candidates for potential DNA mini-barcodes. These markers were chosen for their demonstrated reliability and efficacy in the identification of fungal species. Our analysis highlights their variable effectiveness in species discrimination. In our study, we observed that the ribosomal marker ITS demonstrated fewer issues with PCR amplification compared to the protein-coding marker RPB2. This finding aligns with the observations made by Schoch et al. (2012), who also reported superior amplification success rates for the ITS marker in fungal taxa. Despite the promising role of the RPB2 gene in fungal phylogenetics, our study underlines the necessity for precise and strategic primer design to maximize its potential for species differentiation. Effective use of this gene as a molecular marker is contingent upon selecting genomic regions that not only exhibit sufficient variability but are also representative of the genetic diversity needed to distinguish closely related species.

In fungal DNA barcoding research, the complete ITS region, encompassing ITS1, 5.8S, and ITS2, is commonly recommended as the target area. This region typically provides sufficient information to distinguish between different species, making it a standard choice for fungal species identification due to its considerable sequence variability which is suitable for differentiating species (Schoch et al. 2012). However, when considering the selection of DNA mini-barcodes, often only one of the sub-regions—ITS1 or ITS2—can be utilized due to constraints related to target sequence length, amplification length, and ease of amplification. In this study, ITS-a primers specifically target the ITS1 region, whereas ITS-b and ITS-c primers are designed to amplify the ITS2 region. Although ITS1 and ITS2 are both components of the ITS region, they exhibit differences in sequence variability and effectiveness for species identification. Although ITS1 is generally considered more variable and thus a more suitable species marker, there are instances where ITS2 demonstrates greater variability (Nilsson et al. 2008). Nilsson et al. (2008) have also shown a correlation between the variabilities of ITS1 and ITS2, suggesting that these regions complement each other and do not evolve independently. Furthermore, both markers are recognized as suitable for DNA metabarcoding applications. The ITS region has gained popularity as a molecular marker due to its effectiveness in DNA mini-barcoding and high-throughput sequencing, making it extensively used for detecting and identifying fungal species (Badotti et al. 2017; Garnica et al. 2016; Wang et al. 2019).

In this study, we introduced a set of universal primers, ITS-a, targeting the ITS1 gene sequence, yielding a DNA mini-barcode of approximately 290 bp tailored specifically for the identification of toxic *Amanita* species. The efficacy of this primer set was evaluated through PCR amplification and sequencing of 43 *Amanita* samples. Our findings revealed the efficacy of the DNA mini-barcode in identifying toxic *Amanita* species through NCBI sequence alignment, intra-species variation, and phylogenetic tree analysis. The in vitro digestion experiment on mushroom samples demonstrated the amplification ability of the DNA mini-barcode despite DNA degradation. Comparing conventional DNA barcodes with DNA mini-barcodes for identifying *Amanita* samples post an in vitro digestion experiment, our study found that DNA mini-barcodes outperformed conventional DNA barcodes.

Although barcode length does not strictly dictate the efficacy of species identification, there exists an optimal range of fragment lengths that strikes a balance between informativeness and practical applicability in species identification. When molecular markers of DNA barcodes are too short, identification accuracy declines. PCR amplification can successfully amplify the target sequence when the DNA barcode is less than 150 bp, but the resulting target sequence obtained through direct sequencing is too short to effectively identify the species (Meusnier, et al. 2008). Consequently, an optimal barcode length should be selected to ensure it contains adequate variable sequences to facilitate precise taxonomic resolution. This balance is essential not only for enhancing the robustness of the amplification process but also for ensuring the genetic coverage is comprehensive enough to unambiguously identify species, particularly when dealing with closely related taxa.

Nevertheless, the study’s limitation, represented by the relatively modest number of *Amanita* samples, accentuates the imperative need for expanded research with a more extensive sample size to enhance the statistical robustness of the analysis. Furthermore, exploring the applicability of these findings to other fungal groups using DNA mini-barcodes presents an interesting avenue for future research. The lack of reference sequences for certain *Amanita* species in the GenBank database underscores the ongoing need for sustained endeavors in establishing and updating reference databases to improve identification accuracy using DNA barcodes.

In conclusion, the evaluated DNA mini-barcode emerges as a promising and practical tool for the analysis of processed and digested *Amanita* mushrooms, offering significant potential for optimizing species identification and traceability in instances of mushroom poisoning. The study contributes valuable insights to the field of applied microbiology and biotechnology, advancing our understanding of molecular tools for fungal identification in challenging conditions. However, it is imperative to recognize the limitations associated with our DNA mini-barcoding method. One critical concern is the time-intensive nature of the process from tissue extraction to DNA analysis, which poses challenges in scenarios requiring rapid diagnosis for treating poisoned patients. Integrating our method with rapid diagnostic technologies, such as expedited DNA extraction methodologies and real-time sequencing techniques, could substantially reduce the time required for species identification. Furthermore, the reliance on DNA sequencing instruments presents challenges for on-site sample detection, limiting its immediate applicability in emergency scenarios. Addressing these limitations, future research directions could focus on integrating DNA mini-barcoding with portable rapid detection technologies, such as nanotechnology, to enable swift on-site species identification and enhance the method’s practicality. Additionally, while the DNA mini-barcoding method may not offer immediate treatment options, it can serve as a valuable tool for post-exposure forensic analysis. This aspect is essential for developing targeted treatments and informing public health interventions to prevent future poisonings. By acknowledging these limitations and exploring innovative solutions, the effectiveness and applicability of DNA mini-barcoding in species identification and post-exposure analysis can be significantly enhanced.

## Supplementary Information

Below is the link to the electronic supplementary material.

Supplementary file1 (PDF 263 KB)

## Data Availability

The authors confirm that the data supporting the findings of this study are available within the article and/or its supplementary materials. All generated sequences of *Amanita* were deposited on GenBank. All data generated or analyzed in this study can be obtained from the authors upon reasonable request.
