# Supplementary material for: Using a DNA mini-barcode within the ITS region to identify toxic Amanita in mushroom poisoning cases
Source: Appl Microbiol Biotechnol. 2024 Jun 17;108:376. doi: 10.1007/s00253-024-13219-x (PMC11182838; doi:10.1007/s00253-024-13219-x)

Supplemental Materials

Journal: Applied Microbiology and Biotechnology

Title: Using a DNA mini-barcode within the ITS region to identify toxic *Amanita* in mushroom poisoning cases

The authors: Ran-Ran Xing[a], Wen-Ming Bai[a,b], Di Hu[a,c], Ting-Ting Deng[a], Jiu-Kai Zhang[a], Ying Chen[a, *]

The affiliations of the authors:

[a] National Key Laboratory for Market Supervision (Food Authentication), Chinese Academy of Inspection and Quarantine, No.11, Ronghua South Street, Daxing District, Beijing, 100176, China;

[b] College of Food Science, South China Agricultural University, Guangzhou, 510642, China;

[c] Nanjing University of Finance and Economics, Nanjing, 210046, China.

The e-mail address and telephone of the corresponding author: chenyingcaiq@163.com, 0086-10-53897910.

Table S1. Identification results of Amanita samples based on full-length DNA barcoding (Bai, et al., 2021).

No.	Sample number	Species	Full-length barcode results			
			LSU	ITS	RBP2	*β-tubulin*
1	CAIQ12001	*Amanita sinocitrina*	＋	＋	＋	＋
2	CAIQ12002	*Amanita melleiceps*	＋	＋	＋	＋
3	CAIQ12003	*Amanita rubrovolvata*	＋	＋	＋	＋
4	CAIQ12004	*Amanita caojizong*	*Amanita pseudoprinceps*（99%）	*Amanita pseudoprinceps*（99%）	*Amanita pseudoprinceps*（100%）	*Amanita pseudoprinceps*（100%）
5	CAIQ12005	*Amanita caojizong*	＋	＋	＋	＋
6	CAIQ12006	*Amanita caojizong*	*Amanita yuaniana*（99%）	*Amanita yuaniana*（100%)	*Amanita yuaniana*（100%）	*Amanita yuaniana*（99%）
7	CAIQ12007	*Amanita kotohiraensis*	＋	＋	＋	＋
8	CAIQ12008	*Amanita oberwinklerana*	*Amanita rimosa*（99%）	*Amanita rimosa*（100%）	*Amanita rimosa*（100%）	*Amanita rimosa*（100%）
9	CAIQ12009	*Amanita oberwinklerana*	＋	＋	＋	＋
10	CAIQ12010	*Amanita fuliginea*	＋	＋	＋	＋
11	CAIQ12011	*Amanita fuliginea*	＋	＋	＋	＋
12	CAIQ12012	*Amanita fuliginea*	＋	＋	＋	＋
13	CAIQ12013	*Amanita subglobosa*	＋	＋	＋	＋
14	CAIQ12014	*Amanita pseudogemmata*	＋	＋	＋	＋
15	CAIQ12015	*Amanita virgineoides*	＋	＋	＋	＋
16	CAIQ12016	*Amanita virgineoides*	＋	＋	＋	＋

17	CAIQ12017	*Amanita neoovoidea*	＋	＋	＋	＋
18	CAIQ12018	*Amanita sp.*	*Amanita minutisquama* （99%）	*Amanita minutisquama* （99%）	*Amanita minutisquama* （99%）	*Amanita minutisquama* （100%）
19	CAIQ12019	*Amanita exitialis*	＋	＋	＋	＋
20	CAIQ12020	*Amanita rufoferruginea*	＋	＋	＋	＋
21	CAIQ12021	*Amanita sculpta*	＋	＋	—	N
22	CAIQ12022	*Amanita rimosa*	＋	＋	＋	＋
23	CAIQ12023	*Amanita rimosa*	＋	＋	＋	＋
24	CAIQ12024	*Amanita orientifulva*	—	—	—	＋
25	CAIQ12025	*Amanita flavipes*	＋	＋	＋	＋
26	CAIQ12026	*Amanita subpallidorosea*	＋	＋	＋	＋
27	CAIQ12027	*Amanita subpallidorosea*	＋	＋	＋	＋
28	CAIQ12028	*Amanita vaginata*	＋	＋	N	N
29	CAIQ12029	*Amanita vaginata*	＋	＋	N	N
30	CAIQ12030	*Amanita sphaerobulbosa*	—	—	—	＋
31	CAIQ12031	*Amanita fritillaria*	＋	＋	—	＋
32	CAIQ12032	*Amanita pallidorosea*	＋	＋	＋	＋
33	CAIQ12033	*Amanita pallidorosea*	＋	＋	＋	＋
34	CAIQ12034	*Amanita manginiana*	—	—	—	＋
35	CAIQ12035	*Amanita gymnopus*	—	—	—	＋
36	CAIQ12036	*Amanita sinensis*	＋	＋	＋	＋

37	CAIQ12037	*Amanita sinensis*	*Amanita aspericeps*（99%）	*Amanita aspericeps*（99%）	*Amanita aspericeps*（99%）	*Amanita aspericeps*（99%）
38	CAIQ12038	*Amanita javanica*	＋	＋	—	—

Notes: "+" indicates the sequence matches the given species name on NCBI (⩾99% similarity); "-" indicates the corresponding band was not amplified or the sequencing failed; " N" indicates the sequence does not match in the NCBI (<95% similarity).

Data source: Wenming, B., X. Ranran, C. Liping, P. Tao, L. Hongtao and C. Ying (2021). DNA Barcoding for Identification of Toxic Amanita Species. Food Science 42(4): 9.

Fig. S1. PCR amplification electrophoresis diagram of RPB2 and ITS of *Amanita* samples using different mini-barcode primers. M: DL marker 2000; A1-A2: RPB2-b; B1-B2: RPB2-a; C1-C2: RPB2-c; D1-D2: ITS-a; E1-E2: ITS-b; F1-F2: ITS-c；G1-G2: *β-tubulin*-a；H1-H2: *β-tubulin*-b；1: *Amanita rubrovolvata*; 2: *Amanita caojizong*; b: Blank control.

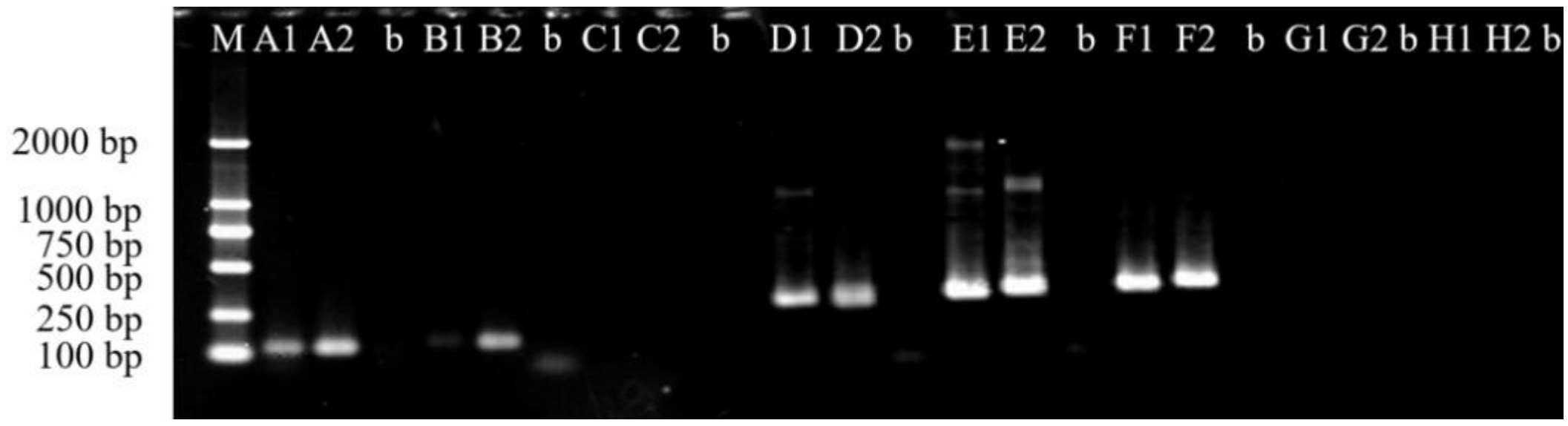

Fig. S2. PCR amplification electrophoresis diagram of *Amanita* samples using RPB2-b and ITS-a primer sets. A: RPB2-b; B: ITS-a; M: DL marker 2000; 1: *Amanita sinocitrina*; 2: *Amanita melleiceps*; 3: *Amanita rubrovolvata*; 4: *Amanita pseudoprinceps*; 5: *Amanita caojizong*; 6: *Amanita yuaniana*; 7: *Amanita kotohiraensis*; 8: *Amanita rimosa*; 9: *Amanita oberwinklerana*; 10: *Amanita fuliginea*; 11: *Amanita fuliginea*; 12: *Amanita fuliginea*; 13: *Amanita subglobosa*; 14: *Amanita pseudogemmata*; 15: *Amanita virgineoides*; 16: *Amanita virgineoides*; 17: *Amanita neoovoidea*; 18: *Amanita minutisquama*; 19: *Amanita exitialis*; b: Blank control.

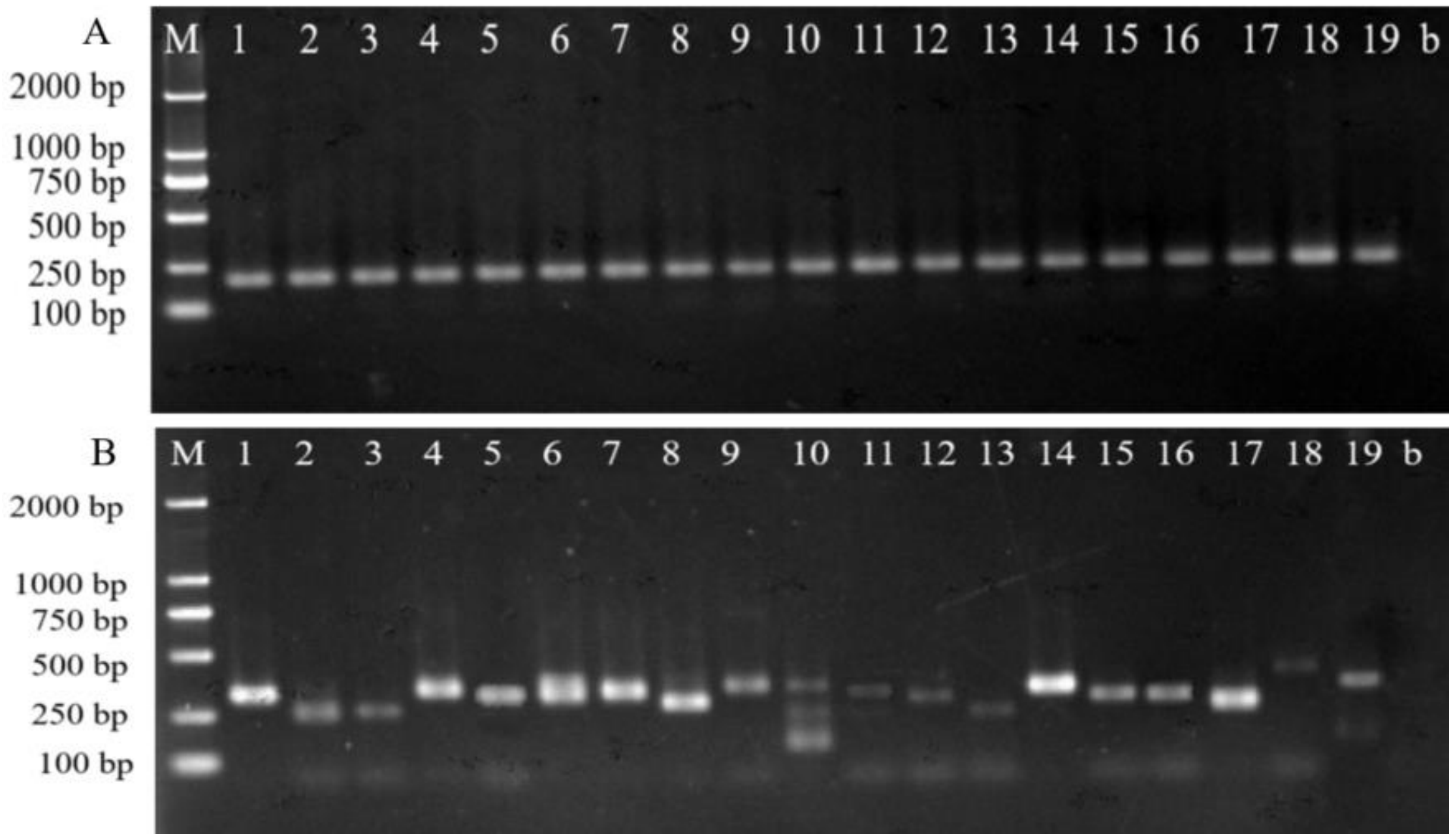

Supplement: Supplementary file 1 [file 253_2024_13219_MOESM1_ESM.pdf]
